# Supplementary material for: Association of company downsizing with the risk of mental distress among employees in the Norwegian working population
Source: PLOS Ment Health. 2025 Jun 24;2(6):e0000247. doi: 10.1371/journal.pmen.0000247 (PMC12798573; doi:10.1371/journal.pmen.0000247)
Supplement: Attachment — Submitted filename: Rebuttal letter.docx [file pmen.0000247.s002.docx]

**Reviewer #1: Rational of the study on the topic could have been further elaborated, and the importance of the study could have been mentioned clearly. Selection of participants in the study, if presented graphically it would have been easier to understand and visualize the sample selection process of the participants. In descriptive statistics section of result, findings could have been presented in fraction or percentage rather than presenting in numbers because it is easier to visualize results. Odds ratio could have been presented to one decimal point. In result table 2, statistically significant p-values could have been highlighted with "*" and bolded as it becomes eye catchy for readers. In discussion section, the probable factors that have favored the association of different factors of downsizing with poor mental health outcomes could have been added for each statistically significant association. The factors associated with, poor mental health outcomes as a result of downsizing could have been compared with similar studies of downsizing and its association with mental health in different context. After the completion of study, recommendation must have been made to protect mental health of employees even though employees go through the downsizing in the organizations. How the finding of the study could be helpful to promote and protect mental health of the employees where downsizing is frequent? How can this study will be helpful for the future researchers?**

Author response: Thank you for taking the time to review the manuscript and for your comments on how to improve the different parts of the manuscript. For clarity, we address the abovementioned questions sequentially below:

**Rational of the study on the topic could have been further elaborated, and the importance of the study could have been mentioned clearly.**

Author response: We appreciate the reviewer’s comment and have now made the contribution of our study more explicit in the revised manuscript. While the relationship between downsizing and employee mental distress has been studied previously, and even more so the relationship between organizational restructuring and employee well-being, our study adds to the literature by using a large and representative sample from three iterations (2013, 2016, 2019) of a nationally representative survey of Norwegian employees, providing robust and generalizable findings. Unlike many previous studies, we account for the employee’s proximity to downsizing within the organization, offering a more nuanced understanding of its impact on mental distress. A key contribution is our inclusion of employees’ self-reported ratings of involvement, information, and support during downsizing, showing that these factors significantly modify the risk of mental distress and suggesting that the process itself—rather than just the fact of downsizing—plays a crucial role in shaping employee well-being. While existing research has often focused on those who lose their jobs, we highlight how downsizing also affects employees who remain, which is particularly relevant given long-standing laws and regulations on employer responsibilities during restructuring that primarily focus on dismissed employees. Our study suggests that ensuring a fair and transparent downsizing process may also be important for the well-being of the remaining employees.

**Selection of participants in the study, if presented graphically it would have been easier to understand and visualize the sample selection process of the participants**

Author response: We have spent some time trying to find the best way to describe the selection of the study participants (and the impact of the different selection criteria) as well as to define a source population and show to what extent the final sample differ from the source population (selection bias). Normally one would define a source population and select participants from this population, but this was feasible in this study, because of the criteria that an individual had to attend two consecutive surveys.

In the revised manuscript, we have replaced table 1 (which showed the selection process) with figure 1. Figure 1 shows the same selection process, but we decided to simplify a little bit by only showing the column from table 2 where 2013-2016 and 2016-2019 are merged. We hope that this provides the reader with a clearer presentation of the selection process.

**In descriptive statistics section of result, findings could have been presented in fraction or percentage rather than presenting in numbers because it is easier to visualize results.**

Author response: We interpret the comment as pertaining to the second paragraph under the title “Descriptive statistics..” in the result section. We agree that percentages are helpful to the reader and perhaps preferable in most cases. In the revised manuscript, we included percentages together with the numbers for the first categorization of respondents into the group of respondents that did or did not experience downsizing, as well as percentages for subgroups with another denominator.

**Odds ratio could have been presented to one decimal points:**

Author response: We agree that this is an option, and we do not have a strong preference. We checked the guidelines for authors and STROBE guidelines to help us decide, but did not find anything on this matter. The most important thing here is to be consistent and not to confuse the reader by using one decimal in the manuscript text and two in the tables. The corresponding author has previously published in other PLOS journals and used two decimal points there. We have therefore kept two decimal points consistently in the revised version; however, we are willing to change this if asked to do so.

**In result table 2, statistically significant p-values could have been highlighted with "*" and bolded as it becomes eye catchy for readers.**

Author response: In the revised version, statistically significant (p < 0.05) HRs with 95 % CI and p-values are highlighted with “*” and bold font in table 2.

**In the discussion section, the probable factors that have favored the association of different factors of downsizing with poor mental health outcomes could have been added for each statistically significant association. The factors associated with poor mental health outcomes as a result of downsizing could have been compared with similar studies of downsizing and its association with mental health in different context. After the completion of study, recommendations must have been made to protect mental health of employees even though employees go through the downsizing in the organizations. How the finding of the study could be helpful to promote and protect mental health of the employees where downsizing is frequent? How can this study will be helpful for the future researchers?**

Author response: As a result of the comments above, as well as comment #3 from reviewer #2, we see that there was a need to thoroughly improve the discussion. We have revised the paragraphs in the discussion under “Comparison with other studies and interpretation”. We now elaborate more on the meaning and interpretation of each individual finding with reference to other relevant studies. Especially, we now relate the findings to the overall literature on restructuring, as well as to the established policies and requirements for organizations performing downsizing and restructuring. We believe this explains better how the findings in this study can be used to prevent negative effects of downsizing.

We would like to add that we were a bit unsure how to respond to the two first questions raised by the reviewer regarding the discussion section. If there are remaining issues that we have not addressed properly here or in the manuscript, we apologize, and hope to get the possibility to do so in another round.

**Reviewer #2: Thank you for giving me the opportunity to review this manuscript.
I thank the authors for a well written and clear manuscript. The methods are clear, the analysis fits the study objectives and the results are easy to follow. While the paper is beautifully written, it can be improved with some minor revisions below.**

Author response: Thank you for taking the time to review the manuscript, for your comments on how to improve the manuscript, and the compliments.

**1. Downsizing and mental distress is widely covered in literature, the authors need to provide a clear and unique contribution of their study to literature. What is new about this study that is not known? This clarity will make the study more valid.**

Author response: We appreciate the reviewer’s comment. This comment was also made by reviewer 1, but we have copied our response under both comments.

We have now made the contribution of our study more explicit in the revised manuscript. While the relationship between downsizing and employee mental distress has been studied, and more so the relationship between restructuring overall and well-being, our study adds to the literature by using a large and representative sample from three iterations (2013, 2016, 2019) of a nationally representative survey of Norwegian employees, providing robust and generalizable findings. Unlike many previous studies, we account for the employee’s proximity to downsizing within the organization, offering a more nuanced understanding of its impact on mental distress. A key contribution is our inclusion of employees’ self-reported ratings of involvement, information, and support during downsizing, showing that these factors significantly modify the risk of mental distress and suggesting that the process itself—rather than just the fact of downsizing—plays a crucial role in shaping employee well-being. While existing research has often focused on those who lose their jobs, we highlight how downsizing also affects employees who remain, which is particularly relevant given long-standing laws and regulations on employer responsibilities during restructuring and downsizing that primarily focus on dismissed employees. Our study suggests that ensuring a fair and transparent downsizing process may also be important for the well-being of retained employees.

**2. Line 45-47 the message is not clear, revision might be needed**

Author response: Thank you for pointing this out. We have revised the sentence to make the message clearer.

**3. While the authors have done a decent job in most parts of the manuscript, the discussion has not been given sufficient attention. As it presently stands, the discussion sounds more of a repetition of results not a deep delve into the meaning of results and an elaborate arrangement of arguments. This section needs to be thoroughly improved. Furthermore, the discussion still lacks depth and a holistic representation of findings.**

Author response: We see the reviewers’ point here and agree that the discussion needed further improvement. Reviewer #1 also commented on the need for improvement. In the discussion of the revised manuscript, in the section “Comparison with other studies and interpretation”, we now elaborate more on the meaning of each finding. We decided also to relate the findings to the overall literature on restructuring, as well as to the established policies and requirements for organizations when performing downsizing and restructuring, which include requirements for providing employee information and involvement to those that lose their job. We have also tried to arrange the section in a manner that helps the reader follow a sequence of findings as warranted by the reviewer. Lastly in the section, we try to provide a holistic perspective on the findings, in line with current research and legislation on restructuring. We have also rewritten the conclusion to reflect these changes. We hope that the reviewer finds the manuscript improved in this regard.
