# Supplementary material for: Association of company downsizing with the risk of mental distress among employees in the Norwegian working population
Source: PLOS Ment Health. 2025 Jun 24;2(6):e0000247. doi: 10.1371/journal.pmen.0000247 (PMC12798573; doi:10.1371/journal.pmen.0000247)
Supplement: Attachment — Submitted filename: Rebuttal_letter_auresp_2.docx [file pmen.0000247.s003.docx]

**Reviewer #1: The revised manuscript is well written and all the comments has been addressed properly. The manuscript can be further processed towards the publication.**

Author response: We thank the reviewer for his/her time and for constructive feedback on the manuscript.

**Reviewer #2: I thank the authors for paying careful attention to the comments and addressing them in an intelligible fashion.
I however invite them to make some small revisions to help strength the manuscript.**

Author response: We would like to thank the reviewer for taking the time to review and critically appraise our manuscript once more and for constructive feedback on how to further strengthen the manuscript.

**1. Line 273, sub-heading "Comparison with other studies and interpretation" should be removed because it is redundant. This is what the discussion section is all about, thus, the heading "Discussion" is sufficient.**

Author response: We agree that the sub-heading is redundant here and have removed it from the revised manuscript.  **2. The discussion has greatly improved but could still be made better. After reporting what was found, the authors have a responsibility to interpret what such a finding means, even in the Norway context if possible. We need to know what such results mean before contrasting with other studies. This has not come out clearly. Take an example. Line 281, "Employees exposed to downsizing had a higher risk of incident mental distress" before relating to literature, how do authors interpret this result? what does it mean? once the meaning is clearly elaborated then contrasting with other studies can come in. This alignment helps the reader understand how authors made meaning out of their findings.**

Author response: We agree that a more elaborate interpretation of the main finding would improve the structure and content of the discussion and have tried to amend this in the revised manuscript. First, we make it clear that there could be an effect of downsizing on mental health and reiterate the main mechanisms that could convey such an effect. Second, to provide the reader with an understanding of what this effect would mean in terms of public health, we extrapolate the risk estimates to all employees in Norway. Third, we also try to help the reader understand what this measurement of mental distress by introducing a staging approach to the classification and treatment of mental disorders, starting from a state of well-being to subclinical symptoms that causes distress and to more specific and severe symptoms that causes severe distress.

We hope the reviewer finds the discussion improved. We did consider whether there was a need for a more elaborate interpretation also of the secondary findings in the discussion not explicitly mentioned by the reviewer, but we decided that this was not required, as we had provided a clear interpretation of those findings in the previous version of the manuscript. However, if the reviewer disagrees or has additional comments, we hope that we can have a chance to address them.
